# 3D printing-assisted triple-vessel *in situ* fenestration combined with a diameter-restricting technique for a complex giant aortic arch aneurysm in an octogenarian: a case report and technical innovation

**DOI:** 10.3389/fcvm.2025.1650003

**Published:** 2026-01-20

**Authors:** Xueshi Yin, Hanlin Chen, Jing Ge, Long Tang, Jianping Liu, Yongheng Zhang

**Affiliations:** 1 The Department of Clinical Medicine, North Sichuan Medical College, Nanchong, Sichuan, China; 2 Department of Cardiovascular Surgery, Suining Central Hospital, Suining, Sichuan, China

**Keywords:** 3D printing, aortic arch aneurysm, octogenarian, triple-vessel *in situ* fenestration, case report

## Abstract

**Background:**

Aortic arch aneurysms involving branch vessels traditionally require open surgery with cardiopulmonary bypass, which poses prohibitive risks for octogenarians with complex comorbidities. This case demonstrates the successful application of total endovascular aortic repair (TEVAR) with three dimensional (3D) printing-assisted triple-vessel *in situ* fenestration and a diameter-restricting technique in an 85-year-old patient with a giant (9.0 cm) aortic arch aneurysm involving the left subclavian artery.

**Case presentation:**

The involvement of the three arch branches (brachiocephalic trunk, left common carotid artery, and left subclavian artery) necessitated precise revascularization. In comparison with traditional *in situ* fenestration, 3D printing-guided *ex vivo* fenestration enabled pre-release stent modeling on a 1:1 aortic arch replica (error <1 mm), allowing anatomically tailored fenestration positioning and eliminating blind puncture-related complications. A proximal stent diameter-restricting technique addressed the challenging anchoring zone gradient (33.6 → 27.3 mm), improving stent apposition and reducing type I endoleak risk. Intraoperative multiaccess reconstruction (femoral/axillary/cervical approach) achieved complete aneurysm exclusion. Postoperative computed tomography angiography on day 4 confirmed patent branches and absence of endoleaks, while 6-month follow-up demonstrated stable stent position and no neurological complications.

**Conclusion:**

This case highlights that TEVAR with 3D printing-assisted *ex vivo* fenestration and a diameter-restricting technique can serve as a viable alternative to open surgery for high-risk octogenarians with complex aortic arch aneurysms, overcoming traditional limitations of *in situ* fenestration while preserving cerebral perfusion. Further studies are warranted to validate this approach in larger populations.

## Background

Aortic arch aneurysms remain a formidable challenge in cardiovascular surgery due to their complex anatomical location adjacent to the brachiocephalic trunk, left common carotid artery, and left subclavian artery, as well as their associated high surgical risks (1). Giant aneurysms (diameter >5.5 cm) frequently lead to fatal complications, including acute rupture, retrograde dissection into the ascending aorta, and compression of the trachea and esophagus, as wall tension and rupture risk increase exponentially (2). Although traditional open surgery achieves anatomical repair, it requires cardiopulmonary bypass and deep hypothermic circulatory arrest, with postoperative complications such as stroke and spinal ischemia posing particular risks to older adult patients with cardiopulmonary dysfunction (3). In recent years, total endovascular aortic repair (TEVAR) has significantly reduced surgical trauma through minimally invasive approaches, yet it faces unique technical challenges in aortic arch applications: (1) the geometric heterogeneity of the three arch branches (including vascular angulation and diameter differences) demands precise fenestration positioning; (2) aortic calcification and tortuosity in older patients often result in stent malapposition and Type I/III endoleaks; and (3) secondary intervention risks, such as retrograde Type A dissection, remain at 2.5%–7.5% in complex cases (4–6).

To overcome these limitations, three-dimensional (3D) printing technology has been synergized with advanced endovascular techniques to enable personalized TEVAR. (1) By reconstructing a 1:1 aortic arch model from thin-slice computed tomography (CT) data, surgeons can simulate stent deployment *in vitro* to prefabricate fenestrations for branch vessels, thereby avoiding the risks of *in situ* fenestration. This approach achieves submillimeter precision in fenestration positioning, as demonstrated by error margins <1 mm in physical models (7, 8). (2) 3D modeling also facilitates preprocedural design of stent modifications, such as proximal stent constriction, to match the gradient of the anchoring zone. This technique enhances stent apposition in tortuous or calcified arteries, reducing type I endoleak risks compared with those associated with standard TEVAR (9). (3) For aneurysms involving three arch branches, 3D printing-assisted planning enables systematic optimization of fenestration spacing and stent curvature, addressing the geometric heterogeneity that complicates traditional TEVAR. In older adult populations, this combined approach offers two principal advantages: (1) minimization of contrast exposure and procedure time through preoperative simulation, thereby reducing cerebrovascular risk (1, 7); and (2) avoidance of open surgery-related trauma, which is particularly critical for octogenarians with vascular fragility and comorbidities. However, while previous studies have evaluated 3D printing in TEVAR for patients aged 60–75 years, data on ultra-older patients (≥80 years) with triple-branch involvement remain scarce. Therefore, this case report aimed to demonstrate the feasibility of 3D printing-assisted TEVAR combined with *ex vivo* fenestration and diameter-restricting techniques in this high-risk subgroup, addressing a critical evidence gap in complex aortic arch repair.

## Case presentation

**Figure 1:**
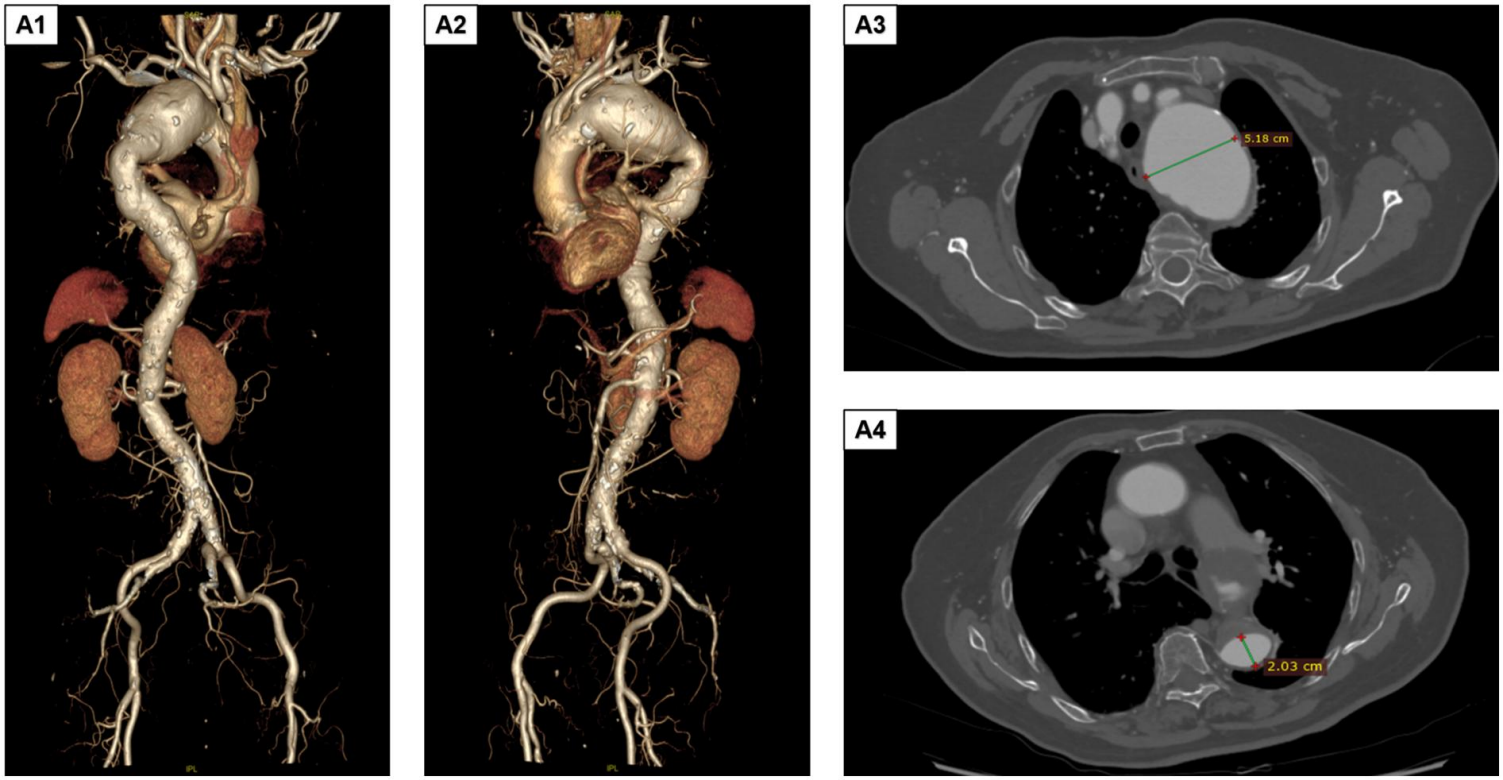
Preoperative DSA and CTA findings. **(A1,A2)** The aortic arch aneurysm involves the left subclavian artery and extends downward to the thoracic aorta. The size of the aneurysmal sac is approximately 9.0 × 5.1 cm. **(A3,A4)** The aortic arch shows aneurysmal dilation, with the maximum diameter measuring approximately a5.18 cm. Additionally, aneurysmal dilation of the thoracic aorta is observed, with a diameter of approximately 2.03 cm. DSA, digital subtraction angiography; CTA, computed tomography angiography.

The patient was an 85-year-old woman who presented with aortic arch aneurysmal dilation discovered during a routine chest CT scan. She reported no clinical symptoms such as chest tightness or dyspnea. Her medical history included hypertension for 4 years (poorly controlled, with admission blood pressure of 161/85 mmHg) and type 2 diabetes for 2 years (random blood glucose level 10.1 mmol/L), with no other systemic diseases. Physical examination revealed no positive findings. Preoperative comprehensive evaluation identified multiple complex vascular pathologies, including lacunar cerebral infarction, moderate stenosis of the anterior and posterior cerebral arteries, vertebral artery stenosis, and atherosclerosis of the coronary and bilateral lower limb arteries. Preoperative digital subtraction angiography and computed tomography angiography (CTA) demonstrated a giant aortic arch aneurysm involving the left subclavian artery and extending into the thoracic aorta (approximately 9.0 × 5.1 cm, Figures 1A1,A2), aortic arch aneurysmal dilation (diameter 5.18 cm), and thoracic aortic aneurysmal dilation (diameter 2.03 cm, Figures 1A3,A4). Considering the patient's advanced age, complex vascular pathology, and high-risk profile for open surgery, TEVAR was selected following multidisciplinary team evaluation.

A detailed, step-by-step protocol for 3D model fabrication and physician-modified stent-graft (PMSG) preparation has been provided as Supplementary Document 1 to facilitate reproducibility. The procedure is summarized in three key phases: (1) Preoperative Planning and Model Generation: segmentation of thin-slice CTA data and 3D printing of a patient-specific, hollow aortic model; (2) Ex Vivo Stent-Graft Modification: *in vitro* deployment on the model, laser-guided fenestration marking, controlled fenestration creation, and cuff reinforcement; and (3) Diameter-Restricting Technique Application: pre-loading and securing of the purse-string suture. The supplementary protocol includes specifics on software settings, printing materials, laser parameters, and suturing techniques.

**Figure 2:**
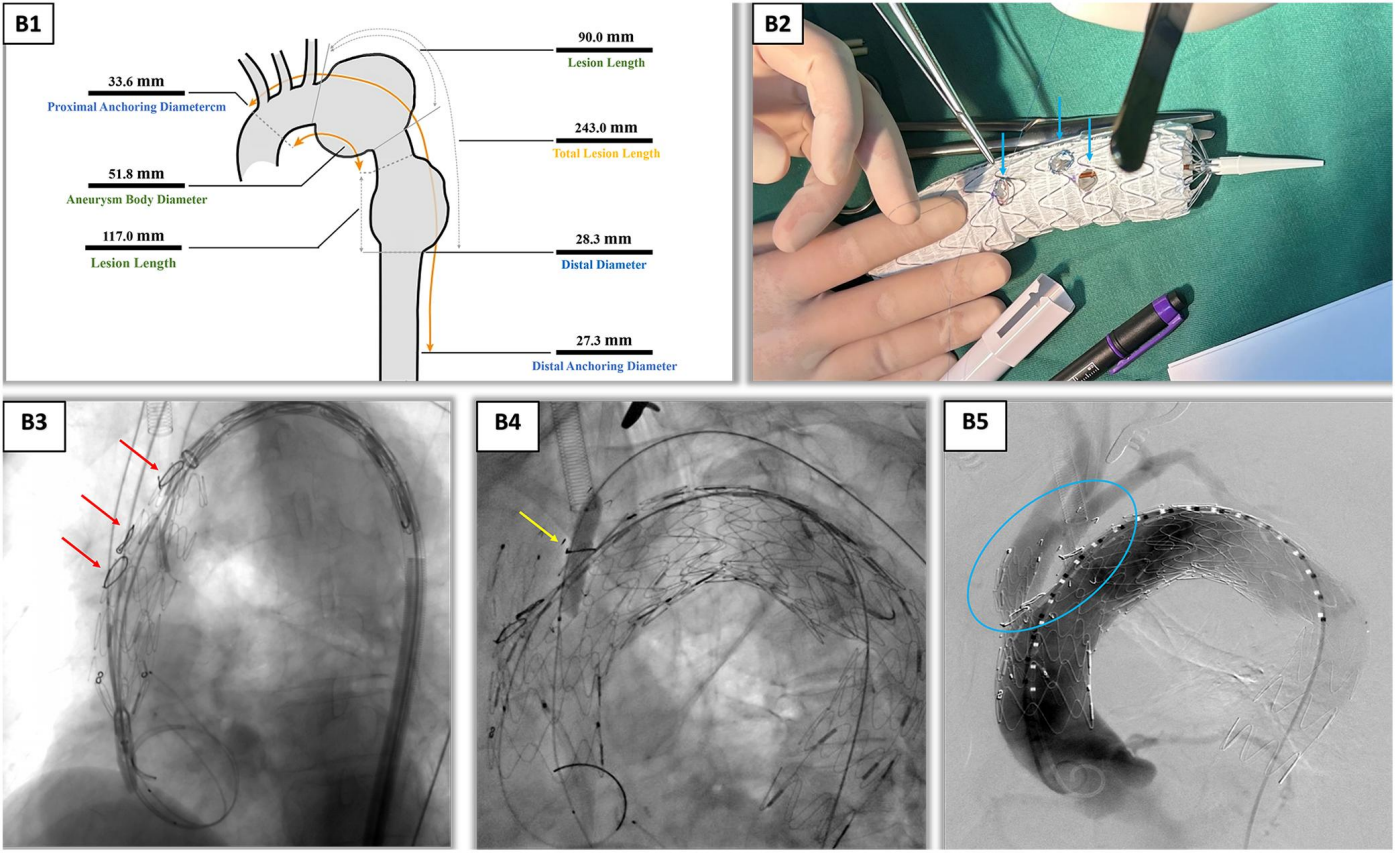
Intraoperative angiography and *ex vivo* stent fenestration modification. **(B1)** Endosize measurement report, providing key vascular parameters for surgical planning. **(B2)** Preoperative planning and stent modification using the patient-specific 3D-printed aortic arch model. This photograph showcases the cornerstone of our technique: the 1:1 hollow aortic arch model, which accurately replicates the patient's anatomy. The stent graft is deployed on this model, allowing for precise ex vivo fenestration. The blue arrows, from left to right, point to the laser-scribed fenestration markers on the stent fabric, which were aligned with the ostia of the left subclavian artery, left common carotid artery, and innominate artery on the model. This process ensured anatomically tailored fenestration positioning and eliminated the risks associated with blind *in situ* puncture. **(B3)** Under DSA guidance, the stent was precisely implanted using coil positioning. The red arrows, from top to bottom, mark the stent implantation positions of the left subclavian artery, left common carotid artery, and innominate artery. **(B4)** Balloon post-dilation of the left subclavian artery branch was performed to ensure good apposition between the stent and vessel wall. **(B5)** After vascular reconstruction, each branch was clearly visualized. Inside the blue circles, the stents in each branch are fully expanded with a good morphology, indicating unobstructed blood flow. DSA, digital subtraction angiography; 3D, three dimensional.

3D reconstruction (Endosize/Mimics) based on thoracoabdominal CTA data (slice thickness, 0.625 mm) yielded the following key parameters: proximal/distal anchoring zone diameters, 33.6/27.3 mm; total lesion length, 243 mm; and interbranch distances, brachiocephalic trunk to left common carotid artery 15.2 mm and left common carotid to left subclavian artery 13.8 mm. Using Geomagic Studio, a 1:1 hollow aortic arch model was fabricated with submillimeter precision. The model's accuracy was validated by comparing key anatomical distances (e.g., interbranch distances) on the model with those on the source CT data, confirming a mean registration error of <0.8 mm (Figure 2B1), serving as a template for *ex vivo* stent modification. A LifeTech TAA3630B200 covered stent was selected and deployed on the 3D model to simulate branch vessel alignment. Fenestration creation was performed on the 3D-printed model and involved: (1) using the model's branch ostia as guides, 5 mm circular markers were laser-scribed on the stent fabric corresponding to the brachiocephalic trunk, left common carotid, and left subclavian artery origins (Figure 2B2); (2) fenestrations were sequentially created with a 5-F electrocautery probe, ensuring smooth edges to minimize intimal injury; and (3) each fenestration was reinforced with a 10 mm Vabahn® polytetrafluoroethylene branch cuff, secured via continuous 7-0 Prolene® suture to prevent stent fabric fraying and enhance sealing. To address the larger diameter of the proximal anchoring zone (33.6 mm), a detachable constriction mechanism was applied: a 4-0 polypropylene suture was pre-loaded through the proximal end of the stent, forming a circumferential purse-string. This degree of constriction (30% reduction) was pre-determined on the 3D-printed model to be the optimal balance between achieving secure apposition at the distal landing zone (resulting in a 15% oversizing) and avoiding excessive radial force on the fragile proximal aorta. When tightened, this reduced the effective stent diameter to 23.5 mm, matching the distal anchoring zone (27.3 mm) with a 15% oversizing margin for optimal apposition. The suture was secured using a sliding knot, allowing intraoperative adjustment during stent deployment.

While the PMSG was being prepared, a second team established multivessel access to the right femoral artery (10F sheath), left brachial artery (6F sheath), left common carotid artery (8F sheath via cutdown), and right axillary artery (8F sheath). Following systemic heparinization (60 mg bolus, maintained at 1 mg/kg/h), a 5F gold-marked PIG catheter was advanced via the left brachial artery to perform aortography, confirming branch vessel anatomy. The femoral access route was used to deploy a Shanghai MicroPort superstiff guidewire, over which the main stent graft was advanced. After positioning the stent at the aortic arch, the proximal purse-string suture was cinched to achieve the pre-planned diameter restriction, followed by sequential deployment of branch stents: (1) brachiocephalic trunk: LifeTech IE-1414-060 self-expanding stent (14 × 14 mm), deployed via right axillary access with angiographic guidance; (2) left common carotid artery: LifeTech PS-C-10040XL covered stent (10 × 40 mm), inserted via left cervical sheath and flared with a 6-mm balloon; and (3) left subclavian artery: same covered stent as that of the carotid, followed by BIOTRONIK Passeo-3510 balloon post-dilation (8 × 40 mm) to ensure cuff apposition. Final angiography confirmed complete aneurysm exclusion with no immediate endoleaks, and the TAA3428B200 stent was deployed distally to extend coverage into the thoracic aorta (Figures 2B3–B5). The total procedure time was 5.40 h. The endovascular deployment and PMSG preparation phases constituted 22% (1.20 h) and 41% (2.20 h) of this duration, respectively. The patient had a blood loss of 300 mL, received a 400 mL transfusion of packed red blood cells, and a total contrast volume of 220 mL was used. Subsequent course included an intensive care unit stay of 22 h, a postoperative hospitalization of 10 days, and an aortic coverage length of 20 cm.

**Figure 3:**
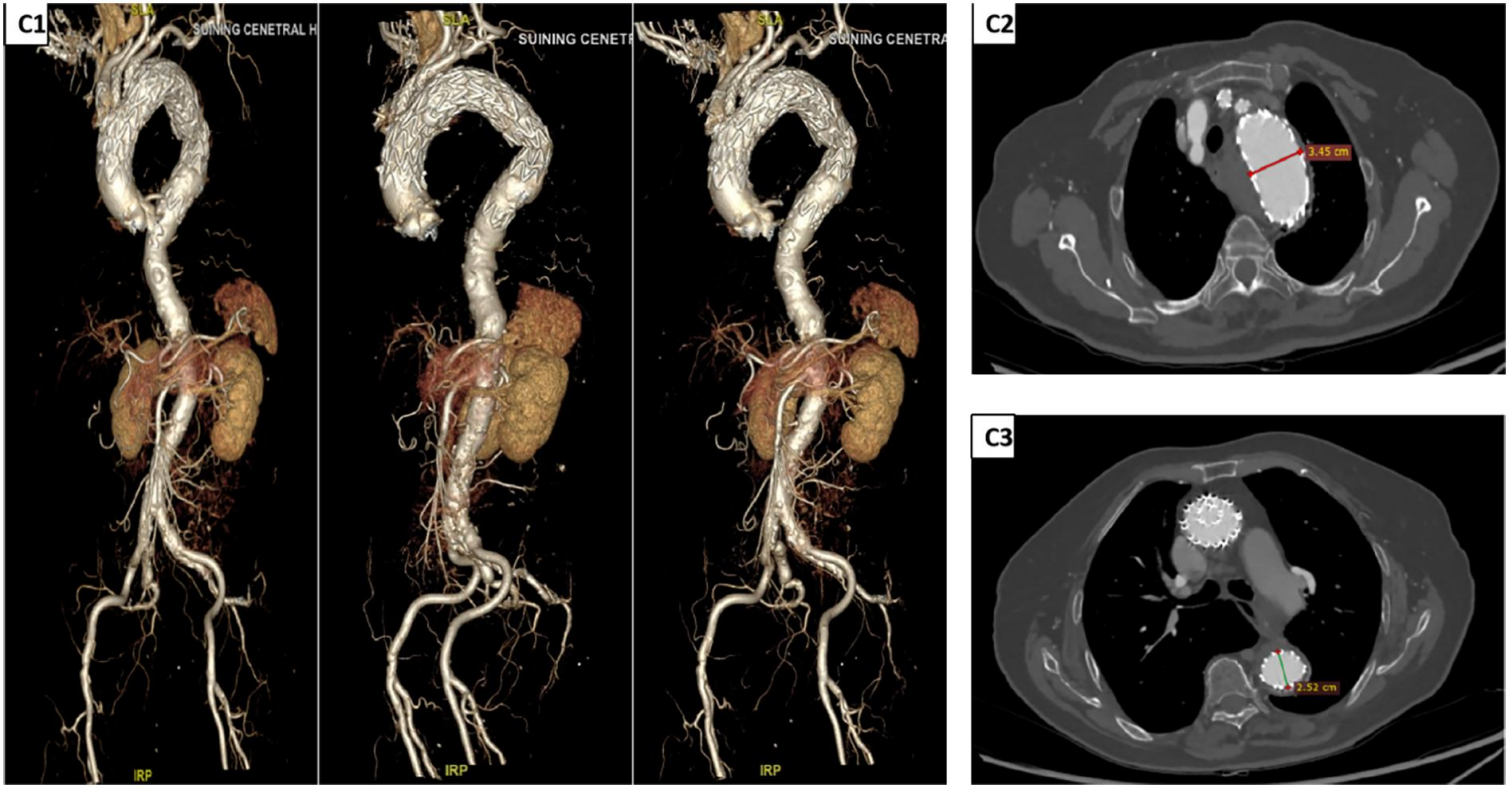
Postoperative DSA and CTA findings. **(C1)** DSA images at different time points after TEVAR (from left to right: 1 week, 1 month, and 6 months post-operation) show that the stent position remains stable, with no evidence of endoleaks. The vascular morphology and hemodynamics are stable. **(C2,C3)** CTA images 1 week after TEVAR. Measurement results indicate that the diameter of the surgical area in the aortic arch is approximately 3.45 cm, and the diameter of the area covered by the TEVAR procedure is approximately 2.52 cm, suggesting a good effect of aneurysm exclusion and a normal process of vascular remodeling. DSA, digital subtraction angiography; CTA, computed tomography angiography; TEVAR, total endovascular aortic repair.

The patient recovered well, with postoperative thoracoabdominal CTA on day 4 demonstrating initial technical success. Discharge occurred on day 10, with stable parameters and no major neurological complications such as paraplegia or stroke. One-month follow-up confirmed stable stent position, patent arch branches, and absence of endoleaks, aneurysm expansion, or retrograde dissection (Figure 3).

## Discussion

Aortic arch aneurysms involving three branch vessels present unique challenges, necessitating a balance between aneurysm exclusion and cerebral perfusion preservation. This challenge is magnified in octogenarians, for whom traditional open surgery carries prohibitive risks of stroke, spinal ischemia, and mortality (1, 3). Here, we demonstrate that a synergistic strategy combining TEVAR with 3D printing-assisted *ex vivo* fenestration and a diameter-restricting technique can overcome these limitations, potentially establishing a new therapeutic paradigm for this high-risk population.

The success of this case hinged on the integration of two key innovations that addressed the core limitations of existing approaches. First, the use of a patient-specific 3D-printed model transformed the fenestration process. By enabling precise, ex vivo prefabrication of fenestrations for all three arch vessels, this approach eliminated the “blind puncture” inherent to *in situ* techniques, which carries an 8%–12% risk of embolic debris and a 15%–20% rate of branch misalignment (10, 11). Our method achieved fenestration positioning within 0.8 mm of target ostia and avoided the need for temporary cerebral shunting, thereby reducing cerebral ischemia time and stroke risk (12). Second, the custom diameter-restricting technique directly solved the critical issue of anchoring zone mismatch (33.6 → 27.3 mm), which exceeds the 5 mm tolerance of standard TEVAR and heightens the risk of type I endoleak (6). The pre-loaded, adjustable purse-string suture allowed for a 30% reduction in proximal stent diameter, creating an optimal 15% oversizing at the distal landing zone. This not only ensured a seal in a calcified, tortuous artery but also mitigated the risk of excessive radial force causing dissection in fragile aged vasculature.

The entirely endovascular nature of this approach provided profound clinical benefits, avoiding the physiological insults of sternotomy, cardiopulmonary bypass, and deep hypothermia—key drivers of multiorgan failure in octogenarians. Consequently, operative time and blood loss were significantly lower than open-surgery benchmarks (3). Furthermore, preoperative 3D simulation reduced contrast usage, minimizing nephrotoxicity, while the *ex vivo* strategy preserved continuous cerebral perfusion, a critical advantage for patients with pre-existing cerebrovascular stenosis (7, 13). When contextualized within the existing literature, our case represents a significant leap. While previous studies have utilized 3D printing in TEVAR for dual-branch aneurysms or in younger cohorts (9, 14, 15), this is the first documented triple-branch reconstruction in an octogenarian. For instance, compared to the hybrid TEVAR approach by Gonzalez-Urquijo et al. (14), which required carotid-subclavian bypass, our pure endovascular method circumvented surgical morbidity entirely. Similarly, while Tong et al. (9) demonstrated the diameter-restricting technique in a younger population, we have validated its safety and efficacy in an ultra-older patient with extreme anatomy.

The integration of TEVAR with PMSG was central to the success of this case. While conventional TEVAR struggles with multibranch aortic arch aneurysms (16, 17), PMSG enables precise reconstruction of the brachiocephalic, left common carotid, and left subclavian arteries through prefenestration and diameter-restricting techniques (18, 19). This approach preserved cerebral and upper limb perfusion while excluding the aneurysm. The diameter-restricting technique improved stent conformity to the aortic arch, mitigating endoleak risk, while radiopaque markers and branch cuffs enhanced positioning accuracy and sealing. Postoperative outcomes were favorable: no neurological complications occurred, and 1-month follow-up confirmed stable stent position, patent branch vessels, and absence of endoleaks or aneurysm progression.

Beyond the immediate technical success, this case underscores a potential paradigm shift in the management of complex aortic arch disease in ultra-older adults. Traditional practice, heavily reliant on open surgery with its inherent risks of stroke and multiorgan failure, often leads to therapeutic nihilism for octogenarians. Our report demonstrates that a fully endovascular, 3D printing-guided strategy can successfully circumvent the need for sternotomy, cardiopulmonary bypass, and deep hypothermic circulatory arrest. This shift moves the treatment goal from simply achieving anatomical repair in operable patients to offering a viable, life-saving intervention for those previously deemed “inoperable.” By mitigating the physiological insults of open surgery, this approach redefines what is possible for high-risk elderly patients, prioritizing minimally invasive recovery and the preservation of quality of life.

To better contextualize our innovation, it is instructive to compare our outcomes with those reported in the existing literature for both advanced endovascular and hybrid techniques, primarily applied to younger populations. For instance, a recent series by Miao et al. on TEVAR using *in situ* fenestration in patients with a mean age of 59 years reported a technical success rate of 97.8% and a 30-day stroke rate of 6.7%. While commendable, their cohort did not include patients over 80 years of age, and their technique retained the risks inherent to *in situ* puncture, such as embolization and branch misalignment (20). In contrast, our 3D printing-assisted ex-vivo approach in an 85-year-old patient achieved technical success while entirely avoiding these specific risks, suggesting a potential safety advantage for fragile, older vessels.

Furthermore, when compared to hybrid arch repair—a common alternative—our purely endovascular strategy demonstrates distinct benefits. Hybrid procedures, which combine open supra-aortic debranching with endovascular stent grafting, eliminate the need for *in situ* fenestration but introduce the morbidity of a surgical incision, cerebral vessel manipulation, and potential for cranial nerve injury. Studies on hybrid arch repair in complex aortic arch pathology patients, such as the work by Liu et al., have reported non-negligible rates of spinal cord ischemia (∼3%) (21). Our case, by achieving complete aneurysm exclusion and triple-branch revascularization without any surgical incision or cerebral bypass, successfully circumvented these specific hybrid-associated complications. This comparison highlights that for selected ultra-older patients, a meticulously planned total endovascular approach may offer a less invasive pathway by avoiding the combined burdens of both full open surgery and the surgical component of hybrid repair.

Despite the promising results, this study has several important limitations that must be acknowledged. First, this is a single-case report from a single center, which inherently limits the generalizability of the findings. The outcomes and technical success described here require validation in larger, multi-center cohorts to establish true efficacy and safety. Second, the approach is highly resource-intensive. It demands (1) specialized expertise in advanced endovascular techniques, including physician-modified stent-graft creation and multi-vessel access management, which has a significant learning curve and limits scalability to high-volume aortic centers; (2) significant infrastructure, specifically access to high-fidelity 3D printing technology and software for model creation, which may not be readily available in all institutions; and (3) increased procedural costs compared to standard TEVAR, attributable to the 3D printing materials, specialized stent grafts, and additional components required for modification. Thus, the diameter-restricting technique's principle is readily transferable to other scenarios with landing zone mismatch, such as complex abdominal aortic aneurysms or type B dissections. Furthermore, collaboration with industry to develop off-the-shelf, customizable stent-graft platforms with integrated constriction mechanisms is a promising pathway to enhance reproducibility and reduce costs. Third, while the short-term follow-up is excellent, the long-term durability of the *ex vivo* fenestrations and the diameter-restricting suture in the high-stress environment of the aortic arch remains unknown. Annual imaging with CT angiography is mandatory to monitor for late complications such as stent fatigue, fabric fraying, suture breakage, endoleak, or aneurysm sac remodeling. Finally, our study lacks formal patient-reported outcome (PRO) measures, such as health-related quality of life assessments. While the clinical success and absence of major complications are unequivocally positive, standardized tools like the EQ-5D or the SF-36 would have provided a more holistic understanding of the procedure's impact from the patient's perspective, particularly regarding functional recovery, pain, and overall well-being. The primary focus of this initial technical report was on feasibility and safety; however, we strongly recommend that future prospective studies on this technique in octogenarians systematically incorporate serial PROs at baseline, 1, 6, and 12 months to truly capture its value in preserving or enhancing the quality of life in this vulnerable population.

## Conclusion

In conclusion, this case provides compelling evidence for a paradigm shift in treating complex aortic arch aneurysms in high-risk octogenarians. The integration of 3D printing-assisted *ex vivo* fenestration and the diameter-restricting technique enables a precise, total endovascular repair that successfully avoids the profound trauma of open surgery and cardiopulmonary bypass. This approach challenges the traditional contraindication of advanced age and complexity, suggesting that futility should be determined by anatomical feasibility and patient-specific planning rather than chronological age alone. While this technique demonstrates compelling short-term feasibility, its long-term durability requires rigorous validation. Prospective multicenter studies with standardized 1-, 3-, and 5-year follow-up endpoints are imperative to monitor for late device-related complications and to definitively establish the role of this approach in the management of complex aortic arch disease. Until such long-term data is available, the application of this technique should be accompanied by a commitment to stringent, lifelong patient surveillance.

## Data Availability

The original contributions presented in the study are included in the article/Supplementary Material, further inquiries can be directed to the corresponding authors.

## References

[1] Cho T Uchida K Yasuda S Onakatomi Y Fushimi K Kaneko S Investigation of risk factors and outcomes of aortic arch aneurysm repair in octogenarians. J Cardiothorac Surg. (2025) 20(1):220. 10.1186/s13019-025-03417-7 40275328 PMC12023417

[2] Senser EM Misra S Henkin S. Thoracic aortic aneurysm: a clinical review. Cardiol Clin. (2021) 39(4):505–15. 10.1016/j.ccl.2021.06.003 34686263

[3] Menges AL Zimmermann A Stoklasa K Reitnauer D Meuli L Reutersberg B. Hospital incidence, sex disparities, and perioperative mortality in open surgically treated patients with aneurysms of the ascending aorta and aortic arch in Switzerland. Healthcare (Basel). (2024) 12(3):388. 10.3390/healthcare12030388 38338273 PMC10855317

[4] Kim KY Byun SJ Yun KH Lee SY Ryu DW Rhee SJ Early experience of thoracic endovascular aortic repair: a local single hospital experience. J Korean Surg Soc. (2012) 82(5):302–5. 10.4174/jkss.2012.82.5.302 22563537 PMC3341479

[5] Piazza M Squizzato F Milan L Miccoli T Grego F Antonello M Incidence and predictors of neurological complications following thoracic endovascular aneurysm repair in the global registry for endovascular aortic treatment. Eur J Vasc Endovasc Surg. (2019) 58(4):512–9. 10.1016/j.ejvs.2019.05.011 31239097

[6] Kudo T Kuratani T Shimamura K Sawa Y. Early and midterm results of thoracic endovascular aortic repair using a branched endograft for aortic arch pathologies: a retrospective single-center study. JTCVS Tech. (2020) 4:17–25. 10.1016/j.xjtc.2020.09.023 34317956 PMC8307048

[7] Verghi E Catanese V Nenna A Montelione N Mastroianni C Lusini M 3D printing in cardiovascular disease: current applications and future perspectives. Surg Technol Int. (2021) 38:314–24. 10.52198/21.STI.38.CV1422 33970475

[8] Patel H Choi P Ku JC Vergara R Malgor R Patel D Application of three-dimensional printing in the planning and execution of aortic aneurysm repair. Front Cardiovasc Med. (2025) 11:1485267. 10.3389/fcvm.2024.1485267 39944658 PMC11814437

[9] Tong Y Qin Y Yu T Zhou M Liu C Liu C Three-dimensional printing to guide the application of modified prefenestrated stent grafts to treat aortic arch disease. Ann Vasc Surg. (2020) 66:152–9. 10.1016/j.avsg.2019.12.030 31917223

[10] Squizzato F Piazza M Forcella E Coppadoro S Grego F Antonello M. Clinical impact and determinants of fenestration to target vessel misalignment in fenestrated endovascular aortic repair. Eur J Vasc Endovasc Surg. (2024) 67(5):765–74. 10.1016/j.ejvs.2023.10.016 37858703

[11] Li X Shu C Li Q He H Li M Wang L Self-radiopaque markers guiding physician-modified fenestration (S-fenestration) in aortic arch endovascular repair. Front Cardiovasc Med. (2021) 8:713301. 10.3389/fcvm.2021.713301 34490376 PMC8417741

[12] Akeret K Buzzi RM Schaer CA Thomson BR Vallelian F Wang S Cerebrospinal fluid hemoglobin drives subarachnoid hemorrhage-related secondary brain injury. J Cereb Blood Flow Metab. (2021) 41(11):3000–15. 10.1177/0271678X211020629 34102922 PMC8545037

[13] Li WD Keyoumu R Wang C Liu Z. 3D Printing-guided endovascular repair of enormous twisted thoracoabdominal aortic aneurysm with branch stenosis and occlusion. Catheter Cardiovasc Interv. (2023) 101(4):813–6. 10.1002/ccd.30578 36740232

[14] Gonzalez-Urquijo M Hosseinzadeh E Aguirre-Soto A Fabiani MA. Stereolithographic (SLA) 3D printing for preprocedural planning in endovascular aortic repair of a thoracic aneurysm. Vasc Endovascular Surg. (2024) 58(3):343–9. 10.1177/15385744231215560 37944002

[15] Fu DS Jin Y Zhao ZH Wang C Shi YH Zhou MJ Three-dimensional printing to guide fenestrated/branched TEVAR in triple aortic arch branch reconstruction with a curative effect analysis. J Endovasc Ther. (2024) 31(6):1088–97. 10.1177/15266028231161244 36942654

[16] Iba Y Minatoya K Matsuda H Sasaki H Tanaka H Oda T How should aortic arch aneurysms be treated in the endovascular aortic repair era? A risk-adjusted comparison between open and hybrid arch repair using propensity score-matching analysis. Eur J Cardiothorac Surg. (2014) 46(1):32–9. 10.1093/ejcts/ezt615 24431168

[17] Okamoto T Yokoi Y Sato N Suzuki S Enomoto T Onishi R Outcomes of thoracic endovascular aortic repair using fenestrated stent grafts in patients with thoracic aortic distal arch aneurysms. Eur J Cardiothorac Surg. (2024) 65(3):ezae062. 10.1093/ejcts/ezae062 38439540

[18] Yang G Zhou M. Use of physician-modified fenestrated stent-graft with wire loop technique for complex aortic arch aneurysm. Ann Vasc Surg. (2021) 77:343–6. 10.1016/j.avsg.2021.05.032 34455051

[19] Liu YM Keyoumu R Shi YH Wang Z Sun LL Liu Z. 3D Parametric surface planar topological guide plate to create a PMSG for the emergency endovascular repair of an aortic arch aneurysm. J Card Surg. (2022) 37(11):3955–7. 10.1111/jocs.16804 35930597

[20] Miao S Gui L Chen H Ma H Yang H Zhang X Midterm outcomes of *in situ* and *in vitro* fenestrated stent-grafts for endovascular repair of aortic arch pathologies. J Endovasc Ther. (2025):15266028251384920. 10.1177/15266028251384920 41222305

[21] Liu Y Zhang B Liang S Dun Y Guo H Qian X Early and midterm outcomes of type II hybrid arch repair for complex aortic arch pathology. Front Cardiovasc Med. (2022) 9:882783. 10.3389/fcvm.2022.882783 35722105 PMC9201486

