# Supplementary material for: 3D printing-assisted triple-vessel in situ fenestration combined with a diameter-restricting technique for a complex giant aortic arch aneurysm in an octogenarian: a case report and technical innovation
Source: Front Cardiovasc Med. 2026 Jan 20;12:1650003. doi: 10.3389/fcvm.2025.1650003 (PMC12864480; doi:10.3389/fcvm.2025.1650003)
Supplement: Supplementary file 1 [file Datasheet1.docx]

**Step-by-Step Protocol for 3D Aortic Model Fabrication and Physician-Modified Stent-Graft Preparation**

This protocol details the technical process for creating a patient-specific 3D-printed aortic model and using it for the ex vivo modification of a stent-graft, specifically for triple-vessel in-situ fenestration and the application of a diameter-restricting technique. Adherence to this protocol is intended to facilitate reproducibility and precision in complex aortic arch endovascular repair.

**Materials Required**

·Software: DICOM viewer, Segmentation software (e.g., Mimics, 3D Slicer), 3D printing preparation software (e.g., 3-Matic, Geomagic Studio).

·Hardware: High-resolution desktop 3D Printer (e.g., based on Stereolithography - SLA or Digital Light Processing - DLP technology).

·Printing Material: Translucent, biocompatible (for model contact), and flexible photopolymer resin.

·Stent-Graft System: Selected thoracic stent-graft (e.g., LifeTech TAA3630B200).

·Modification Tools: Low-power laser marker (or surgical marker), 5-French electrocautery probe, Vabahn® stent graft or PTFE membrane for cuffs, 7-0 Polypropylene suture, 4-0 Polypropylene suture.

**Phase 1: Preoperative Planning and 3D Model Fabrication**

**Step 1.1: Optimal CT Image Acquisition**

·Obtain a high-resolution, electrocardiogram-gated CTA of the thoracoabdominal aorta.

·Critical Parameters: Slice thickness ≤ 0.625 mm. Ensure the scan covers the entire aortic arch from the ascending aorta to the distal descending thoracic aorta with adequate opacification of the true lumen.

**Step 1.2: Image Segmentation and 3D Model Generation**

1. Import: Load the DICOM data into segmentation software.
2. Thresholding: Apply a Hounsfield Unit threshold to isolate the contrast-filled aortic lumen and the three arch branch vessels (Brachiocephalic Trunk, Left Common Carotid Artery, Left Subclavian Artery).
3. Region Growing & Editing: Use the "Region Growing" tool to select the aortic lumen. Manually edit the mask to remove calcifications, thrombus, and adjacent structures (e.g., pulmonary arteries, bones) that may interfere with model accuracy.
4. Hollow Model Creation:

·Create a 3D object from the edited mask.

·Using the "Shell" or "Hollow" function, generate a 2.0-2.5 mm wall thickness for the model to simulate the aortic wall and provide mechanical stability.

·Ensure the ostia of the three branch vessels are clearly patent and serve as open conduits.

**Step 1.3: 3D Printing and Post-Processing**

1. File Preparation: Export the finalized, hollowed 3D model as an STL file. Import it into the 3D printer slicing software.
2. Printing Orientation: Orient the model to minimize support structures inside the aortic lumen and on critical areas like the branch vessel ostia.
3. Printing: Use a translucent, flexible resin. Print according to manufacturer specifications.
4. Post-Processing:

·Carefully remove the model from the build platform and detach all support structures.

·Clean the model thoroughly with isopropyl alcohol to remove residual resin.

·Post-cure the model according to the resin manufacturer's guidelines to achieve optimal mechanical properties.

**Step 1.4: Model Validation**

·Use digital calipers to measure key distances on the physical model (e.g., interbranch distances, aortic diameters at landing zones).

·Compare these measurements against the same distances measured in the source segmentation software.

·Acceptance Criterion: A mean registration error of < 1 mm is required for clinical use.

**Phase 2: Ex Vivo Stent-Graft Modification**

**Step 2.1: In-Vitro Stent Deployment on Model**

**·**Mount the 3D-printed aortic model securely in a fixture that allows easy access to all sides.

·Under fluoroscopic guidance in a simulated setup, deploy the selected stent-graft (e.g., TAA3630B200) inside the hollow model, ensuring it is positioned in the intended final deployment location.

**Step 2.2: Laser-Guided Fenestration Marking**

**·**Using the branch vessel ostia on the 3D model as a direct visual guide, employ a low-power laser marker to ablate a 5-mm circular marker onto the stent-graft fabric at the exact center of each intended fenestration (BT, LCCA, LSA).

·Alternative: If a laser is unavailable, use a sterile surgical marker to make precise marks, acknowledging a potential for minor smudging.

**Step 2.3: Controlled Fenestration Creation**

**·**Remove the stent-graft from the model.

·Using a 5-French electrocautery probe set to a pure cutting current at 20W, carefully puncture the center of each laser-scribed marker.

·Gently enlarge the fenestration to a diameter of 5-6 mm by moving the probe in a circular motion. Ensure the edges are smooth and free of frayed fabric.

**Step 2.4: Fenestration Reinforcement**

**·**Cut a 10-mm circular cuff from a Vabahn® stent-graft or a PTFE membrane.

·Align the cuff over the created fenestration on the external surface of the main stent-graft.

·Using a continuous 7-0 Polypropylene suture, secure the cuff to the main stent-graft fabric. Place 5-6 stitches per quadrant to ensure a robust, hemostatic seal and prevent fabric fraying.

**Phase 3: Diameter-Restricting Technique Application**

**Step 3.1: Purse-String Suture Placement**

·At the proximal end of the stent-graft (the end destined for the larger-diameter anchoring zone), pre-load a 4-0 Polypropylene suture in a purse-string fashion.

·The suture should pass through the stent graft fabric and the first stent apex, creating a complete circle.

·Use a sliding knot (e.g., Roeder's knot) that can be tightened intraoperatively but remains secure under tension. Leave long ends for manipulation.

**Step 3.2: Pre-Procedural Sizing and Adjustment**

**·**On the 3D-printed model, cinch the purse-string suture and deploy the stent-graft to confirm the desired final diameter reduction (e.g., 30% in the presented case) is achieved and provides optimal apposition in the distal landing zone.

·Mark the suture ends to indicate the target cinching point for the actual procedure.

**Quality Control and Sterilization**

·Visually inspect the entire modified stent-graft for any defects, loose sutures, or misaligned fenestrations.

·Perform a final fluoroscopic check to ensure radiopaque markers are intact and the device functions as intended.

·The final Physician-Modified Stent-Graft (PMSG) must be sterilized according to the institutional protocol for implantable devices (e.g., Ethylene Oxide gas sterilization) before clinical use.
